# Efficacy of Novel Carbon Nanoparticle Antioxidant Therapy in a Severe Model of Reversible Middle Cerebral Artery Stroke in Acutely Hyperglycemic Rats

**DOI:** 10.3389/fneur.2018.00199

**Published:** 2018-04-09

**Authors:** Roderic H. Fabian, Paul J. Derry, Harriett Charmaine Rea, William V. Dalmeida, Lizanne G. Nilewski, William K. A. Sikkema, Pitchaiah Mandava, Ah-Lim Tsai, Kimberly Mendoza, Vladimir Berka, James M. Tour, Thomas A. Kent

**Affiliations:** ^1^Department of Neurology, Baylor College of Medicine, Michael E. DeBakey VA Medical Center, Houston, TX, United States; ^2^Department of Neurology and Center for Translational Research on Inflammatory Diseases, Baylor College of Medicine, Michael E. DeBakey VA Medical Center, Houston, TX, United States; ^3^Department of Chemistry, Rice University, Houston, TX, United States; ^4^Division of Hematology, Department of Internal Medicine, The University of Texas Health Science Center at Houston, McGovern Medical School, Houston, TX, United States; ^5^Department of Neurology, Baylor College of Medicine, Houston, TX, United States; ^6^Departments of Chemistry, Computer Science, Materials Science and NanoEngineering, Smalley-Curl Institute and the NanoCarbon Center, Rice University, Houston, TX, United States

**Keywords:** diabetes mellitus, stroke, rat model, hyperglycemia, antioxidants, nanomedicine, transient middle cerebral artery occlusion

## Abstract

**Introduction:**

While oxidative stress can be measured during transient cerebral ischemia, antioxidant therapies for ischemic stroke have been clinically unsuccessful. Many antioxidants are limited in their range and/or capacity for quenching radicals and can generate toxic intermediates overwhelming depleted endogenous protection. We developed a new antioxidant class, 40 nm × 2 nm carbon nanoparticles, hydrophilic carbon clusters, conjugated to poly(ethylene glycol) termed PEG-HCCs. These particles are high-capacity superoxide dismutase mimics, are effective against hydroxyl radical, and restore the balance between nitric oxide and superoxide in the vasculature. Here, we report the effects of PEG-HCCs administered during reperfusion after transient middle cerebral artery occlusion (tMCAO) by suture in the rat under hyperglycemic conditions. Hyperglycemia occurs in one-third of stroke patients and worsens clinical outcome. In animal models, this worsening occurs largely by accelerating elaboration of reactive oxygen species (ROS) during reperfusion.

**Methods:**

PEG-HCCs were studied for their protective ability against hydrogen peroxide in b.End3 brain endothelial cell line and E17 primary cortical neuron cultures. *In vivo*, hyperglycemia was induced by streptozotocin injection 2 days before tMCAO. 58 Male Sprague-Dawley rats were analyzed. They were injected IV with PBS or PEG-HCCs (4 mg/kg 2×) at the time of recanalization after either 90- or 120-min occlusion. Rats were survived for up to 3 days, and infarct volume characteristics and neurological functional outcome (modified Bederson Score) were assessed.

**Results:**

PEG-HCCs were protective against hydrogen peroxide in both culture models. *In vivo* improvement was found after PEG-HCCs with 90-min ischemia with reduction in infarct size (42%), hemisphere swelling (46%), hemorrhage score (53%), and improvement in Bederson score (70%) (*p* = 0.068–0.001). Early high mortality in the 2-h in the PBS control group precluded detailed analysis, but a trend was found in improvement in all factors, e.g., reduction in infarct volume (48%; *p* = 0.034) and a 56% improvement in Bederson score (*p* = 0.055) with PEG-HCCs.

**Conclusion:**

This nano-antioxidant showed some improvement in several outcome measures in a severe model of tMCAO when administered at a clinically relevant time point. Long-term studies and additional models are required to assess potential for clinical use, especially for patients hyperglycemic at the time of their stroke, as these patients have the worst outcomes.

## Introduction

Based on many lines of evidence, oxidative stress is a major pathophysiological factor in ischemia and reperfusion injury. This evidence is exemplified by robust neuroprotection in multiple transgenic antioxidant overexpression models of ischemia/reperfusion (1, 2). However, no clinical trial of antioxidant therapy in any form of brain injury has shown benefit (3, 4). We believe this failure is due to two major factors: (1) There are severe limitations in currently available antioxidants that hinder their effectiveness when employed *following* ischemia as opposed to pretreatment (5) and (2) oxidative stress injury is quantitatively more important under specific clinical circumstances, so a benefit might be missed if it is not tested under the most relevant conditions. In stroke, those conditions are typically those that have the worst outcomes such as *hyperglycemia* at the time of stroke when treated with recanalization therapy (6).

Several defense mechanisms exist to cope with oxidative radicals generated during normal physiology (2, 7, 8). These mechanisms consist of enzymes and other proteins that modify the radical species in a series of steps ultimately leading to water. For example, the fate of superoxide radical $\left({\text{O}}_{2}^{•-};\text{SO}\right)$ when dismutation catalyzed by superoxide dismutase (SOD) is to generate the intermediate unstable molecules (e.g., hydrogen peroxide; H_2_O_2_) or new radicals (hydroxyl; ^•^OH) that can be generated by this process as H_2_O_2_ encounters iron as a catalyst through the Fenton reaction (9). Under normal conditions, there are sufficient levels of protective proteins for detoxification. Under pathological circumstances, however, these protective factors are depleted. After acute injury, they cannot upregulate fast enough. As a result, unstable intermediates are formed that become part of a radical cascade leading to damage and disruption of a wide variety of vital functions.

Given these considerations, once a radical cascade begins, we previously summarized the limitations of many current antioxidants (5) including the following: (A) mechanism of action: many antioxidants “transfer” the radical to another unstable species. SOD generates H_2_O_2_ that can subsequently generate ^•^OH. Under normal circumstances, catalase, and glutathione are in sufficient quantities to quench the resultant radicals. This may not be the case under pathological conditions; SOD may actually generate more damaging species, (B) need for regeneration: many antioxidants, such as vitamin E and vitamin C, require regeneration (10) and require factors (glutathione) that are themselves consumed in the oxidative milieu, (C) limited capacity: most current antioxidants have limited capacity and are unlikely to be able to cope with a burst of radicals and their subsequent unstable products if administered after the burst is initiated. High dose albumin, recently failing to show benefit as an antioxidant in stroke (11), has a restricted number of thiol moieties that quench radicals (12) and (D) selectivity: high selectivity is a disadvantage if the agent’s mechanism involves radical transfer and depends on downstream enzymes to cope with newly formed radicals. Nearly, every currently available antioxidant shares one or more of these limitations (5).

For this study, we have selected a condition that predicts a poor outcome in stroke: transient cerebral ischemia in the face of hyperglycemia at the time of the stroke. These circumstances are associated with increased expression of oxidative radicals (13–15). The kinetics of SO production is highly relevant to clinical outcomes in stroke. Our laboratory has previously studied this time course in a normoglycemic rat model of transient middle cerebral artery occlusion (tMCAO), using a cytochrome c-coated electrode on the cortical surface which detects SO release. In the case of normoglycemia, the SO radical is only released upon the onset of recanalization after occlusion time >90 min (13). Importantly, 90 min is considered an early time point that could be used widely to start catheter-based recanalization therapy. Longer time to recanalization is associated with declining benefit (16) and higher mortality after unselected endovascular procedures (17). Notably, hyperglycemia accelerates and magnifies oxidative burst in tMCAO (14) and worsens outcome in acute stroke models and stroke patients, especially those who receive recanalization therapy (18–20) by increasing mortality and hemorrhagic transformation (6). Hyperglycemic animal models demonstrate poor reflow, enhanced edema, higher mortality, and hemorrhagic conversion (14, 21–23), particularly with longer or more severe ischemia before recanalization (15, 6, 22).

In our reanalysis of the NINDS rt-PA dataset, patients with large artery stroke appear to be most susceptible to hyperglycemia when undergoing thrombolytic therapy (24, 25). In an earlier review, we concluded that poorer outcome is likely due to generation of a pro-thrombotic, pro-inflammatory, and vasospastic vascular phenotype (6). Variability of glucose may be a major contributing factor following clinical stroke (26). Notably, treatment of hyperglycemia after onset of stroke does not appear to improve outcomes (27, 28), although a definitive trial has not yet been performed specifically in the acute stroke setting. With a lack of a proven neuroprotectant therapy for hyperglycemic stroke, new approaches are needed for this especially vulnerable subgroup of patients especially in the context of newer recanalization therapies in which diabetes, glucose variability or hyperglycemia *per se* predicts a lower percentage of patients with a good outcome and/or higher likelihood of hemorrhagic transformation (29–32).

In this study, we employ a novel class of antioxidants to address the oxidative imbalances seen following tMCAO under conditions in which oxidative stress is quantitatively more important. Soon after the discovery of carbon-based buckministerfullerenes (C_60_) (33), these nanomaterials were shown to have antioxidant characteristics (34). Subsequent modifications and applications to models of injury identified neuroprotective properties (35) but also a low threshold for further modification lest their antioxidant capacity be reduced (36). Subsequent generation of a series of different carbon nano-formulations by our research group (37) identified a formulation of carbon nanoparticles (38) that addresses the limitations of current antioxidants described earlier. Through a series of experiments, first in cell-free systems, then in tissue culture and finally *in vivo*, we identified a class of carbon nanoparticles that we term hydrophilic carbon clusters (HCCs) as highly effective antioxidants (39) with unique potential as *in vivo* therapeutics (40–43). We specifically demonstrated their superior efficacy to two clinical failed antioxidants, poly(ethylene glycol) (PEG)-SOD and the precursor to NXY-059 (4), phenyl butyl nitrone, PBN (41) in culture by their ability to reduce the damaging effect of the mitochondrial toxin, antimycin A, when given AFTER the toxin, while pretreatment was needed for the other agents. These particles are small (40 nm in length, 1–2 nm in diameter, comparable to a hydrated protein), highly functionalized to generate hydrophilic moieties with the addition of PEG to provide solubility in biological fluids, stable at room temperature and without apparent toxicity after systemic injection seen thus far (41).

Here, we report *in vitro* and *in vivo* evidence that hydrophilic carbon clusters, conjugated to poly(ethylene glycol) (PEG-HCCs) can mitigate major detrimental effects of oxidative stress. In tissue culture, we demonstrate that PEG-HCCs are able to mitigate the detrimental effects of H_2_O_2_ even though administered after the addition of the H_2_O_2_. *In vivo*, we show that PEG-HCCs administered intravenously at a clinical relevant time (onset of recanalization) can mitigate detrimental effects of the hyperglycemia.

## Materials and Methods

The protective effects of PEG-HCCs in cell culture were tested using the murine brain endothelial cell line, bEnd.3 (44). This cell line was selected because of the delayed effects of transient ischemia at the neurovascular unit that impair reperfusion and promote edema (6). Experiments with neuronal cells were also performed with E17 murine cortical neurons. Oxidative injury rescue experiments were performed with 100 µM H_2_O_2_ because it achieved approximately 50% cell death after 24 h in bEnd.3 cells.

### Culture of bEnd.3 Cells

bEnd.3 cells (ATCC) were grown in T-75 (75 cm^2^) flasks containing Dulbecco’s modified Eagle’s medium (4 mM l-glutamine adjusted to contain 1.5 g/L sodium bicarbonate and 4.5 g/L glucose, 90%; fetal bovine serum, 10%) (Atlanta Biological) in an incubator. Aliquots of 30,000 cells in 0.1 mL were added directly onto sterile 24-well plates. The cells were allowed to attach for 15 min after which an additional 0.9 mL of media is added before the cells are placed in an incubator and allowed to grow for 48 h.

### Hydrogen Peroxide Protection by PEG-HCCs in bEnd.3 Cells

Cultured bEnd.3 cells were then treated with either PBS as a control or 100 µM hydrogen peroxide both with and without PEG-HCC (8 mg/L) added after 15 min. After all the additions, the cultures were incubated at 37°C in 5% CO_2_ overnight. The Live/Dead assay (calcein AM/ethidium homodimer-1) (Cat #L3224, ThermoFisher) was performed per the manufacturer’s instructions and the number of live cells are counted using a Nikon eclipse 80i microscope set to the FITC channel.

### Culture of E17 Murine Cortical Neurons

E17 primary murine cortical neurons (A15586, ThermoFisher) were seeded on to a poly-d-lysine coated 48-well plate at a density of 50,000 cells/well in 500 µL of neurobasal (Cat #21103, ThermoFisher) media containing 1× B-27 supplement and 100 µM GlutaMAX (Cat #35050, ThermoFisher). The neurons were incubated overnight at 37°C in 5% CO_2_. The following morning, 250 µL of media in each well was exchanged with fresh complete media. Afterward, 250 µL of media was replaced twice on days 4 and 7.

### CellROX ROS Formation Assay

A 10 mM solution of H_2_O_2_ was prepared by diluting 51 µL of 9.8 M H_2_O_2_ in sterile water. Two wells containing 50,000 neurons were left as untreated controls, two wells were treated with 47 µL of 85 mg/L PEG-HCCs, and 250 µL of media was removed from four wells and replaced with 250 µL of 100 µM H_2_O_2_ in complete media. After 15 min, 47 µL of 85 mg/L PEG-HCCs was added by pipette, gently mixed, and incubated for 30 min. Simultaneously, 6 mL of a 10 µM solution of CellROX Deep Red (C10422, ThermoFisher) was prepared in complete Neurobasal media by the addition of 24 µL of 2.5 mM CellROX Deep Red dye. After incubating the neurons for 30 min, 250 µL of media was removed from each well and was replaced with 250 µL of 10 µM CellROX Deep Red solution and incubated for an additional 30 min at 37°C with 5% CO_2_. The neurons were rinsed twice by first removing 400 µL of media from each well and gently adding an additional 400 µL of warmed PBS. Finally, 400 µL of the media was removed and replaced with 4% formaldehyde in PBS and fixed for 30 min at 4°C in a refrigerator.

The fixed neurons were imaged at 20× magnification using a Nikon Eclipse Ti equipped with a Photometrics CoolSNAP HQ2 sensor and a 670 nm emission filter (Cy5). Phase contrast images and 670 nm fluorescence images were collected of each well. The average fluorescence signal from each cell was calculated by including only the fluorescence originating from the area of the cell soma. Average cellular fluorescence was normalized to the untreated control cells.

### Cytotoxicity Assay

Due to greater sensitivity to hydrogen peroxide, we tested both 50 and 100 µM H_2_O_2_ in plated neurons. PEG-HCCs were added right after the H_2_O_2_ and cells incubated overnight. Live/Dead assay was performed as above and live cells counted.

### *In Vivo* Testing in Hyperglycemia tMCAO Model

We utilized tMCAO and the filament model (45) in the context of acute hyperglycemia following streptozotocin injection (22, 46). We selected this method of generating hyperglycemia because acute hyperglycemia as a stress reaction in non-diabetics is associated with particularly poor outcomes (47) and less elevation in glucose is needed to increase poor outcomes in non-diabetics compared with diabetics (48).

### Synthesis and Characterization of PEG-HCCs

The carbon core of the PEG-HCCs is prepared by subjecting purified (removing exogenous carbon black and gross metal contaminants) single-walled carbon nanotubes (SWCNTs) to a harsh oxidation procedure which uses fuming sulfuric acid (excess SO_3_, oleum) and nitric acid (38, 39). Nitric acid initiates the oxidation and cutting process which both shortens the SWCNTs to ~35–40 nm and splits them to remove any tubular residues, thus generating shortened oxidized HCCs. Harsh acidic conditions dissolve and remove even trace metal contaminants as determined by inductively coupled plasma mass spectrometry. The surface of the HCCs is functionalized with various oxygen-containing moieties such as alcohols, ketones, and carboxylic acids, rendering the HCCs water soluble in spite of their many remaining hydrophobic domains. Characterization details including infrared spectroscopy (FTIR), Raman spectroscopy, X-ray photoelectron spectroscopy, atomic force microscopy, thermogravimetric analysis, UV–vis spectroscopy, dynamic light scattering, and zeta potential can be found in Berlin et al. (42).

### Induction of Hyperglycemia and tMCAO

All procedures were approved by the Baylor College of Medicine IACUC and the Michael E. DeBakey VA Medical Center R&D Committee. Outcome measurements were performed by coauthors blinded to expected outcomes (William V. Dalmeida and Harriett Charmaine Rea). Rats were rejected from subsequent analysis based on the surgeon’s assessment of peri-procedural errors or procedure related death, concomitant illness (e.g., respiratory compromise) or mechanical dysfunction, with the surgeon blind to their quantified outcomes.

Male Sprague-Dawley rats weighing 330–350 g were delivered to the vivarium 1 week before experiments to allow for acclimation. Hyperglycemia was induced by injecting sterile filtered streptozotocin 60 mg/kg IP. Control rats are injected with sterile filtered normal saline. Two days later rats were subjected to tMCAO using the filament model as published (22). They were fed with standard chow and water *ad lib* and exposed to a standard light–dark daily cycle. In preparation for the MCA occlusion, rats were deeply anesthetized in an induction chamber with 3% isoflurane followed by intubation and mechanically ventilated with 2.0–2.5% isoflurane in an oxygen:air mix of 30:70. The tail artery was cannulated using sterile technique with PE-50 polyethylene catheters for monitoring mean arterial blood pressure, blood pH, PCO_2_, PO_2_, glucose, as well as additional blood chemistries. The tail vein was then cannulated with a 24-gage 0.75″ angiocath to administer an infusion of intravenous fluids. A rectal temperature probe was used, and the temperature maintained at 37 ± 0.5°C with a heating pad. Vitals such as O_2_ saturation, heart rate, average CO_2_, and total CO_2_ were monitored throughout the surgery. Analgesics were administered sub Q during the procedure to alleviate postsurgical pain and were continued postsurgery. Ketaprofen, an NSAID, was injected subcutaneously at 5 mg/kg in addition to buprenorphine at a dose of 0.05–0.1 mg/kg. Atropine was injected subcutaneously if needed at 0.054 mg/kg.

Without pausing anesthesia, focal cerebral ischemia was induced by occluding the origin of the MCA using the intraluminal suture insertion method. Rats were inverted following induction of anesthesia and an area of skin over the right carotid artery was prepped by clipping hair and scrubbing with betadine, rinsing with alcohol, and painting with iodine. An incision was made over the carotid bifurcation, and the carotid bifurcation was exposed with blunt dissection. The internal carotid artery and pterygopalatine artery were ligated at the origin. A small incision is placed in the internal carotid through which a 0.25 mm nylon monofilament is introduced. Initially, the filament was threaded up the internal carotid exactly 1.7 cm from the bifurcation to occlude the middle cerebral artery. Anesthesia was maintained through the duration of MCA occlusion. The filament was then withdrawn, and the internal carotid artery was ligated distal to the arterial incision. During ischemia, a warming blanket was used to maintain body temperature through the duration of MCA occlusion while the animal’s body temperature is sustained with the heating pad. Rats were weaned off the respirator, observed and kept warm until alert and recumbent, and then returned to their cages.

Postsurgical animals received a soft, purified maintenance diet of 31M Diet Gel along with a 98% sterile water polymer HydroGel. In addition, moistened rat chow was placed at the bottom of the cage. Following surgery, blood was collected from rats every morning for glucose levels. Hyperglycemic rats were given NPH Lente insulin to keep glucose levels under 250 mg/dL. Overall, 21% of rats in the 90-min group and 22% of rats in the 120-min group were given insulin on day 0 and day 1, respectively. Pain medications ketaprofen and buprenorphine were continued daily postsurgery once or twice a day, respectively. Rats were observed twice a day for signs of distress and/or pain and euthanized if they meet the criteria.

### Administration of PEG-HCCs or PBS Control

PEG-HCCs were injected at a dose of 4 mg/kg (volume <0.1 mL) in a tail vein just before suture removal. Injection was repeated after 2 h. This dosing protocol was derived initially from concentrations that were maximally protective against various toxins *in vitro* culture and transformed into *in vivo* dosing based on estimated blood volume of distribution in rats. The dosing was confirmed as beneficial and well tolerated from that successfully used in mild traumatic brain injury complicated by hemorrhagic hypotension and repeated in 2 h based on the approximate 2-h blood half-life observed in our normal mice experiments (40, 41).

### Neurological Function (Bederson’s Score)

A behavioral assay, a modified Bederson test was used for acute disability assessment on post-op day 3 (49). Neurological function is assessed from 0 (normal) to 6 at the end of the 3-day period. Either spontaneous death or euthanasia due to undue stress was scored 7. The scoring was as follows:

(1) Rats are first suspended by their tails and reaching for a table by the forelimbs was observed. Rats will normally reach for the table with both limbs, a score of 0. A score of 1 is assessed if only one limb is used to reach for the table.
(2) The rat is then placed on a rough surface which he can easily grab onto with his paws when given a gentle push on the shoulder. A score of 0 is a strong grasp on the rough surface with good resistance when pushed. If slight resistance seen in one paw, a score of 1 is assessed. If the rat offers no resistance at all when pushed in one direction a score of 2 is assessed.
(3) The last test is an observation of rats in an enclosed area (18″ × 36″) where the rat is free to roam. A score of 0 is assessed if the rat can walk the entire length of the enclosure without circling. A score of 1 is given if the rat walks the entire length of the enclosure and also circles. Animals that only circle and cannot walk the length of enclosure is assessed a score of 2. Rats with major deficits that do not move much when placed in the enclosure is assessed a score of 3. The sum of assessment scores from each task is used as the final assessment score.

### Infarct Volume, Hemisphere Swelling

At the end of 72 h or at the time of early euthanasia, the rats were euthanized with 150 mg/kg of Nembutal IP. Rats were perfused transcardially with 100 mL of 0.9% saline. The brains were removed and immediately frozen at −20°C for 20 min and then sectioned into 1 mm thickness using a rotary hand microtome. The ischemic damage was evaluated by immunohistochemical analysis using 2% tetrazolium chloride (TTC) staining. Brains sections were incubated in 2% TTC in phosphate-buffered saline for 10 min in the dark at 37°C. Sections were fixed in buffered 4% paraformaldehyde pH 7.4 and photographed. After 30 min, the brains were sectioned into 10, 1 mm-thick slices from anterior to posterior. The area of non-stained infarct in each slice was measured using NIS Elements AR software (Nikon). Non-stained infarct areas of ischemic and control hemisphere areas were calculated and then multiplied by slice thickness and summed. Hemispheric swelling was assessed by the ratio of ischemic and contralateral hemisphere volume. This ratio was used to adjust infarct volume for edema modified from McBride et al. (50) and also served as hemisphere swelling index.

### Hemorrhage Assessment

Several rats undergoing tMCAO suffered a hemorrhage. The hemorrhages were assessed by visual examination of TTC-stained coronal sections for each animal. The hemorrhage was documented by notating which areas in the brain were specifically affected as striatum and/or cortex, the number of sliced sections that intracranial bleeding had occurred, and the intensity of the hemorrhage seen in the affected areas such as petechial or confluent. Further evaluation entails scoring the hemorrhage by quantifying the size of area that was affected as follows: 0—no hemorrhage, 1—single petechial hemorrhage, 2—multiple petechial hemorrhages, 3—single confluent subcortical hemorrhage, and 4—hemorrhages including cortical region.

### Statistical Analysis

Cytotoxicity and cell fluorescence was tested by comparing means and SDs employing Student’s *t*-test. For *in vivo* studies, baseline conditions and ordinal outcome measures were performed by Student’s *t*-test. Proportions were tested using chi-square adjusted for small *n*. Outcome measures were those prospectively defined (infarct volume, hemisphere swelling index, hemorrhage score, and modified Bederson score) and tested by Mann–Whitney *U* non-parametric test due to the potential for non-normally distributed outcomes with the small *n*. Mortality was recorded.

## Results

### *In Vitro* Protection Against H_2_O_2_

**Figure 1:**
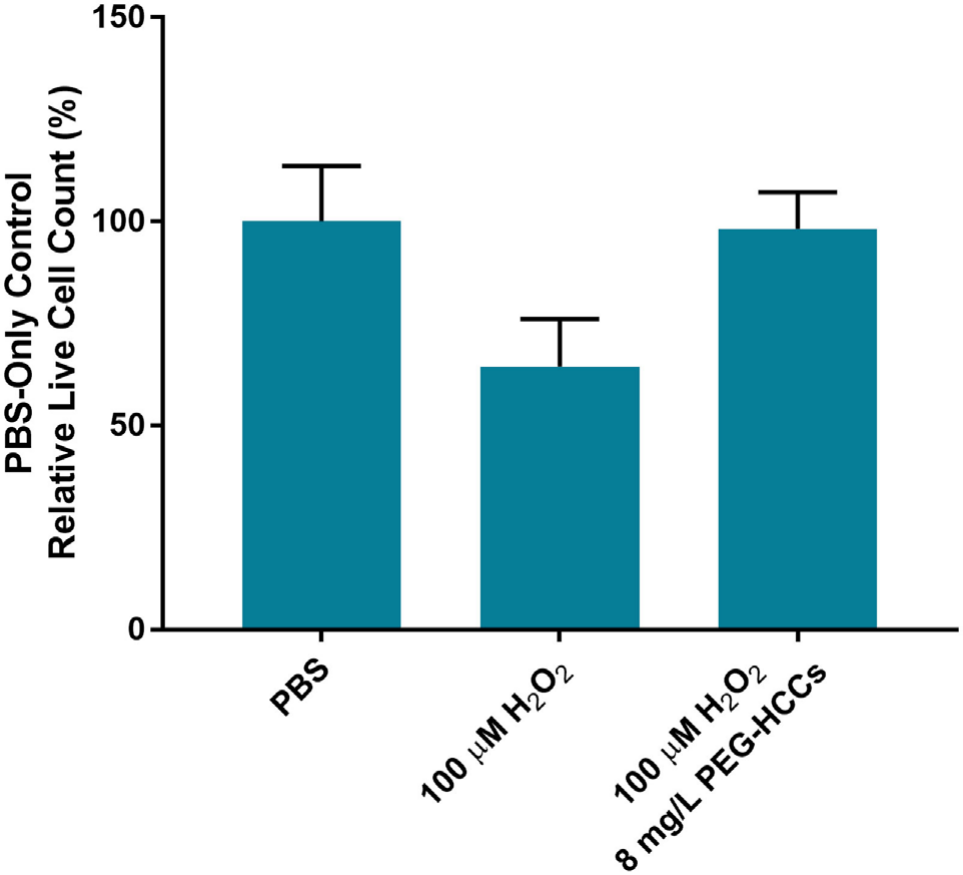
Cell viability following addition of hydrogen peroxide to cultured brain endothelial cells (b.End3). Live cell counts (Live/Dead cell viability assay) per well is presented on *y*-axis as mean and SD of replicates. 100 µM H_2_O_2_ was added and 15 min later either media or hydrophilic carbon clusters, conjugated to poly(ethylene glycol) (PEG-HCCs) (8 mg/mL) was added and live cell/well assessed the following day. H_2_O_2_ reduced cell viability by 50%, which was completely restored by PEG-HCCs.

**Figure 2:**
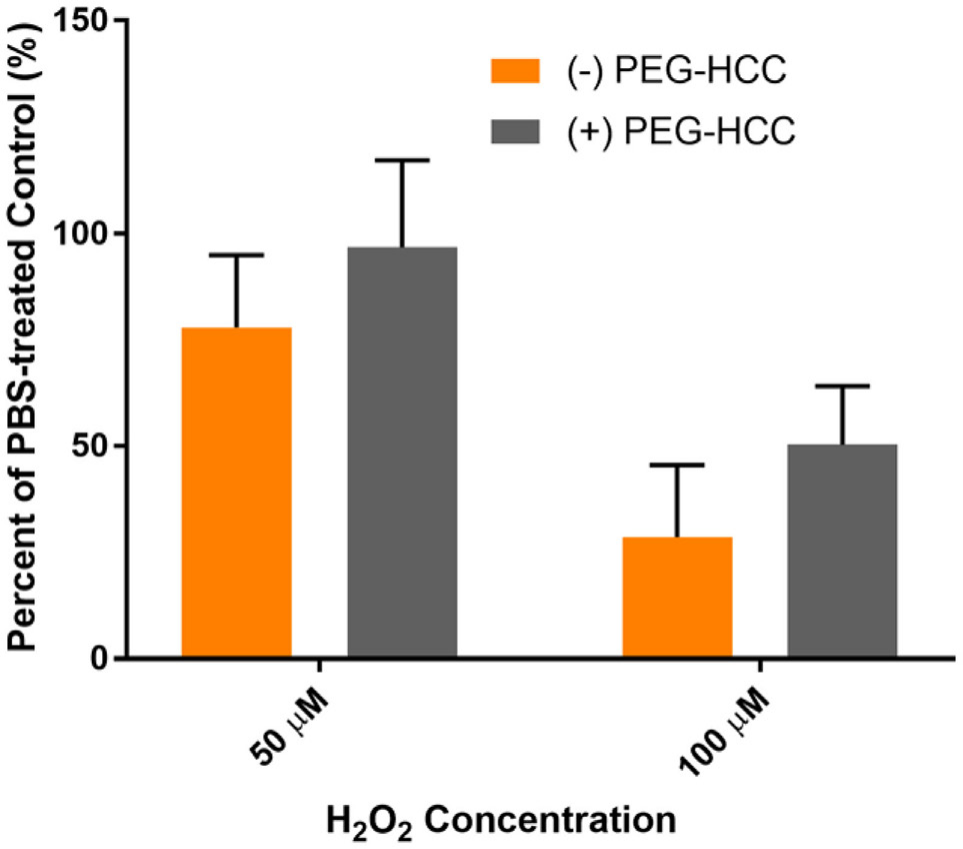
Hydrophilic carbon clusters, conjugated to poly(ethylene glycol) (PEG-HCCs) reduce cytotoxicity of H_2_O_2_ on treated MCNs. PEG-HCCs given at a concentration of 8 mg/L treated immediately following exposure and overnight incubation reduce cell death restored cell number to baseline following 50 µM H_2_O_2_ and doubled cell count following the much more toxic 100 μM H_2_O_2_.

The protection of PEG-HCCs against hydrogen peroxide was measured in both cultured murine cortical endothelial bEnd.3 cells and in cultured primary murine cortical E17 neurons. We observed that 100 µM H_2_O_2_ reduced cell viability in bEnd.3 cells at 24 h by approximately 50% as indicated by a Live/Dead assay (Figure 1). The addition of PEG-HCCs after 15 min restored cell number to baseline (*p* < 0.001 vs H_2_O_2_). In E17 neurons, we found that 100 µM H_2_O_2_ was more lethal in neurons than b.End3 cells, nevertheless, partial restoration was achieved with posttreatment with PEG-HCCs (Figure 2).

### CellROX ROS Assay in E17 Neurons

**Figure 3:**
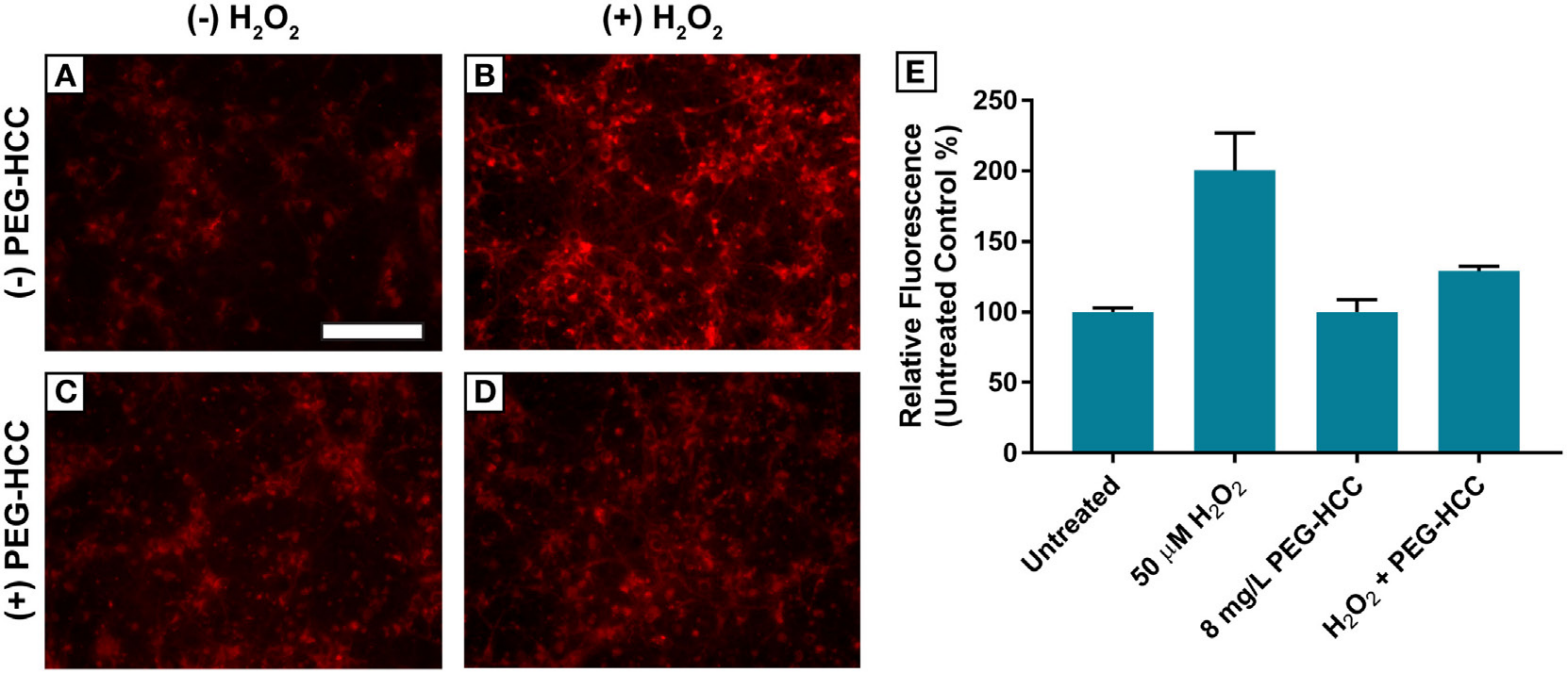
Hydrophilic carbon clusters, conjugated to poly(ethylene glycol) (PEG-HCCs) reduce the oxidation of CellROX fluorescent dye in primary murine cortical neurons by hydrogen peroxide. **(A)** MCNs (50,000 cells/well) untreated. **(B)** MCNs treated with 50 µM H_2_O_2_ for 45 min. **(C)** MCNs treated with 8 mg/L PEG-HCCs for 45 min. **(D)** MCNs treated with 50 µM H_2_O_2_ for 15 min followed by the addition of 8 mg/L PEG-HCCs for an additional 30-min exposure. **(E)** Untreated control normalized fluorescence of oxidized CellROX dye. Total cell counts per condition: untreated (*n* = 137), 50 µM H_2_O_2_ (*n* = 158), 8 mg/L PEG-HCC (*n* = 150), and H_2_O_2_ + PEG-HCC (*n* = 139).

ROS formation was measured using a CellROX assay in cultured murine neurons (Figure 3). E17 cells treated with PEG-HCCs showed no increase in CellROX fluorescence compared with the untreated control (100.1 ± 8.8%). Cells treated with 50 µM H_2_O_2_ for 15 min showed a significant increase in CellROX fluorescence (200 ± 26.5%). Treatment of MCNs with 8 mg/L PEG-HCCs following 15 min of H_2_O_2_ exposure for 30 min showed an increase in CellROX fluorescence of 129 ± 3.4% but was smaller than with H_2_O_2_ by itself. Cell viability was reduced at 50 µM H_2_O_2_ by 20% and was fully restored by PEG-HCCs treatment.

### *In Vivo* tMCAO

Seventy-two rats underwent the procedure. Fifty-eight met criteria for outcome analysis. In the 90-min occlusion, four PBS- and one PEG-HCC-treated rats were excluded, and in the 120-min occlusion group, seven PBS- and two PEG-HCC-treated rats were excluded, primarily for early illness/mortality or procedural problems identified by the operator before assessment of outcomes.

**Figure 4:**
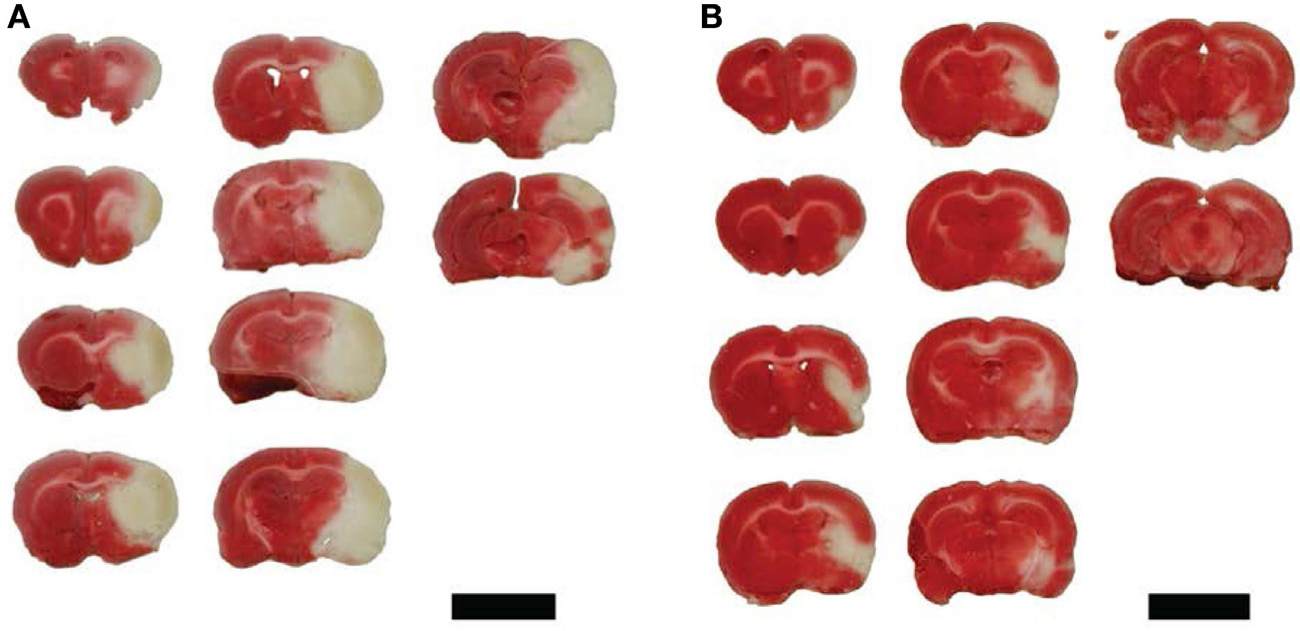
Representative tetrazolium chloride sections demonstrated infarct volume with PBS control treatment and hydrophilic carbon cluster, conjugated to poly(ethylene glycol) (PEG-HCC) treatment following 90-min ischemia and reperfusion. **(A)** PBS control demonstrating entire MCA territory infarction. **(B)** Following treatment with PEG-HCCs and demonstrated considerable cortical sparing. Tissue section groups came from individual rats. Scale bars are 1 cm.

The target of 300 mg/dL preoperative glucose was achieved in the 90-min group. PBS-treated rats showed complete MCA territory infarction (Figure 4A) while PEG-HCCs treated rats showed mostly subcortical infarctions (Figure 4B). Quantification of outcome measures demonstrated that PEG-HCC treatment improved infarct volume, hemorrhagic conversion, hemisphere swelling and Bederson score, with a trend toward reduced mortality (Table 1).

**Table 1:** Results of hydrophilic carbon cluster, conjugated to poly(ethylene glycol) (PEG-HCC) treatment compared with controls in hyperglycemia after 90 min occlusion and assessment at the end of experimental period.

	PBS (*n* = 17)	PEG-HCC (*n* = 16)	*p*-Value
Glucose (mg/dL)	274 ± 69	299 ± 67	0.35
pO_2_	145 ± 19.9	144 ± 19.8	0.92
pCO_2_	40.2 ± 3.15	40.1 ± 5.99	0.96
pH	7.33 ± 0.038	7.34 ± 0.061	0.68
Lesion volume (mm^3^)	275 ± 52	161 ± 84	0.03*
Hemisphere volume change (relative)	12 ± 4.5%	6.5 ± 5.1%	0.027*
Hemorrhage score	1.75 ± 1.16	0.83 ± 0.88	0.068
Mortality rate	5/17	1/16	0.175
Modified Bederson score	3.6 ± 1.5	1.51 ± 0.97	0.001*

The mean overall survival was 2.8 days. Groups did not differ with respect to baseline glucose just before tMCAO or in blood gas parameters taken from a sample of each group. All outcomes were in the direction of improvement with PEG-HCC treatment compared with controls. *P < 0.05.

Survival was markedly diminished at the 120-min time point in the PBS-treated controls, such that no rats survived the day of procedure at the original target glucose (300 mg/dL). We subsequently reduced the streptozotocin dosing until we achieved a target of 200 mg/dL glucose at the onset of the tMCAO procedure. Survival without apparent discomfort to at least 24 h marginally improved in the PBS-treated controls. However, this limited the information that we could obtain from the control group and we did not pursue this time point to full completion. Rats that required sacrifice before 12 h postprocedure were not assessed for infarct characteristics as we felt this would be unreliable. In this time point, we observed positive trends in all measures, with significance achieved in the infarct volume (Table 2).

**Table 2:** Results of hydrophilic carbon cluster, conjugated to poly(ethylene glycol) (PEG-HCC) treatment compared with controls in hyperglycemia after 120 min occlusion and assessment at the end of experimental period.

	PBS (*n* = 14)	PEG-HCC (*n* = 11)	*p*-Value
Glucose (mg/dL)	199 ± 42	203 ± 46	0.900
pO_2_	151 ± 12.6	149 ± 12.2	0.737
pCO_2_	40.9 ± 4.18	43.1 ± 7.38	0.447
pH	7.36 ± 0.047	7.32 ± 0.033	0.056
Lesion volume (mm^3^)	259 ± 121	130 ± 87	0.034*
Hemisphere volume change (relative)	ND	ND	
Hemorrhage score	ND	ND	
Mortality rate	9/14	3/11	0.111
Modified Bederson score	4.8 ± 2.4	2.1 ± 1.8	0.055

The mean overall survival was 2.1 days. Glucose targets were lowered to improve survivability of the procedure. Groups did not differ with respect to baseline glucose just before tMCAO or in blood gas parameters from a representative sample except for trend toward lower pH in the PBS group. All outcomes were in the direction of improvement with PEG-HCC treatment compared with controls with significance achieved with modified Bederson Score. ND: not done because of premature termination of the experiment (see text). *P < 0.05.

## Discussion

In this report, we demonstrated that PEG-HCCs could improve cell survival in both tissue culture models of oxidative injury from H_2_O_2_, particularly in a brain endothelial cell line, an important target of hyperglycemia in stroke. From our CellROX assay on cultured neurons we can conclude that PEG-HCCs prevent the formation of oxidative radicals which would otherwise react with the non-fluorescent CellROX dye to produce a fluorescent derivative.

We also found that treatment with PEG-HCCs at a clinically relevant time point could improve several important features related to stroke outcome in a rat model of tMCAO complicated by acute hyperglycemia. Given that hyperglycemia has major influences on outcome in tMCAO through a dysfunctional vasculature (6), we speculate that the *in vitro* effects are indeed relevant to this *in vivo* protection, which is supported by benefit on two vascular measures: hemisphere swelling and hemorrhagic transformation. The dramatic worsening of outcome with hyperglycemia especially at 2 h in our hands was mitigated to some extent even in this severe condition by administration of PEG-HCCs.

There are several limitations of our study. There are different methods of inducing hyperglycemia that each encapsulate some aspect of both the acute and chronic effects of diabetes and/or hyperglycemia. We selected this acute model because analyses suggest that hyperglycemia in patients without prior diabetes have the worst outcomes (20). Here, we employed short survival periods, which was necessitated by the poor outcomes in the control group. An alternative strategy will be implemented in the future to look at the limits of occlusion time possible with PEG-HCCs without a concomitant delay in recanalization for a comparison control group given the severity of the injury (51). We selected only male rats for this proof of principle study and will need to address sex and age differences before expectation that these results can be clinically translated. In a different carotid occlusion model and hyperglycemia, female sex was associated with less severe outcomes (52). Clinically there are reported differences in both stroke risk and outcomes in diabetics related to gender (53, 54), which is complicated by different risk factors, stroke etiologies, and treatment responses but remains an important issue to address in preclinical models.

The occlusion method has some limitations as well. Endovascular therapy for ischemic stroke has been now shown to be overall beneficial even at longer time intervals in patients who maintain good collateral circulation and when using a new generation of removable stent retrievers (55). While not certain, these improved outcomes could be due to improved recanalization rates as well as less endothelial injury in the process. The suture model approximates some of the features of removable stent-retriever mechanical thrombectomy, but the principle is quite different including application of a removable stent. Use of analgesics and anti-inflammatory agents postprocedure was needed because of the severity of the insult; however, it is not clear what affect these may have had if they interacted with the PEG-HCCs. Also, the severity of the insult likely resulted in a relatively high percentage of subjects excluded (19%), although how this compares with other similar studies is not known since this number is not universally reported. We acknowledge that testing of our materials in larger animal models and more clinically realistic methods of inducing occlusion would be necessary before clinical translation. Nevertheless, the profound worsening by hyperglycemia in this model may model a worst-case scenario of endothelial occlusion/injury that suggest PEG-HCCs may be promising when used in combination with endovascular therapy.

The mechanism of worsened vascular outcomes in these models can be potentially explained at least in part by “uncoupling” of nitric oxide synthase (NOS) (46, 56–58), a phenomenon in which NOS dysfunction, often after oxidation of the cofactor, tetrahydrobiopterin, prevents proper coupling between the oxidase and reductase domains of NOS leading to generation of a SO radical in lieu of reducing l-arginine to NO and citrulline. *In vivo*, there are many potential sources of SO to initiate this effect [e.g., NADPH oxidase (14, 59)]. We have termed an overall increase in SO relative to NO as “functional uncoupling” since the net result, including the toxic product peroxynitrite, is similar regardless of the sources (46, 60). Consistent with this concept, we recently showed that, while both acute hyperglycemia and tMCAO individually cause functional uncoupling in the vasculature in the peri-infarct region, tMCAO with hyperglycemia had a 10-fold synergistic increase in SO relative to NO, still evident at 24 h (46). Persistent oxidative imbalance provides a potential late target for intervention, since the peri-infarct region is critical in mediating many of the vascular complications of stroke (61) such as edema and hemorrhage. The role of oxidative stress is partially supported by recent studies on post-recanalization beneficial effects of uric acid in a hyperglycemic mouse model as well (62).

Hydrophilic carbon clusters, conjugated to poly(ethylene glycol) are a unique antioxidant (39, 63, 64). While the antioxidant potency per carbon atom of PEG-HCCs is within an order of magnitude of prototype antioxidants such as Trolox and vitamin C, the capacity per particle is remarkable. Quantitative electron paramagnetic resonance (EPR) indicates the quenching effect of PEG-HCCs is equivalent to the total SOD activity in human spinal cord (39). Using EPR spectroscopy, and oxyhemoglobin, cytochrome *c*, and pyrogallol red decomposition assays we found that PEG-HCCs convert SO to O_2_, making them ideal for treating ischemia/reperfusion (39). Turnover numbers (moles of consumed SO/moles of PEG-HCCs) were 1 million at physiological pH. Nanomolar concentrations of PEG-HCCs showed typical Michaelis–Menten kinetics with turnovers in the same range as that obtained from the EPR. The catalytic turnover number is about an order of magnitude higher than most efficient single active site enzymes and suggests that a PEG-HCC could possess multiple catalytically active sites. Furthermore, 2.4 nM of PEG-HCCs are able to scavenge 2.8 and 53.7 µM of SO and of ^•^OH, respectively. PEG-HCCs do not quench NO radicals and had no direct effect on peroxinitrate anion (ONOO^−^). Given that NO is constantly produced *in vivo*, is freely diffusible and PEG-HCCs efficiently scavenge SO, this upstream scavenging effect will likely also decrease the amount of ONOO^−^ produced *in vivo*. Taken together, these studies demonstrate that PEG-HCCs address each of the hypothesized limitations of current antioxidants (5): capacity, selectivity, and quenching toxic intermediates. Prior work indicates rapid endothelial cellular uptake, although the mechanism is not yet identified (40).

While modern endovascular procedures show improved outcomes, many patients still do not fully recover. The precise role of reperfusion injury is controversial in those patients who do not recover. Several analyses of the most recent generation of endovascular therapies for the most part find an association of either diabetes *per se*, hyperglycemia or glucose dysregulation associated with poorer outcomes and/or hemorrhagic transformation (29–32). Indeed, even in a stent-retriever study, reanalysis that found that hyperglycemic patients did derive benefit, hyperglycemic patients were 36% less likely to have a good functional outcome, had nearly double the mortality and a fourfold increase in hemorrhagic transformation compared with non-hyperglycemic patients who received the catheter-based therapy (65).

While the presence of “reperfusion injury” remains controversial, we have suggested that it is most likely to be found in those patients that have a concomitant factor such as hyperglycemia or other sources of inflammation (66), and that an important factor to consider in the patients who do not recover are baseline factors that worsen outcome, of which hyperglycemia is a major consideration (6, 51, 67, 68). The abundant preclinical evidence supports that reperfusion oxidative stress is most prominent in this population, so we would expect strategies such as ours to be most effective in this group, if at all. Additional studies are warranted to in clinically relevant animal models encompassing a range of pathologies to address their suitability as an adjunct to recanalization therapies.

## Ethics Statement

All procedures were approved by the Baylor College of Medicine Institutional Animal Care and Use Committee and the Michael E. DeBakey VA Medical Center R&D Committee.

## Author Contributions

Animal handling—RF and HR. *In vitro* cell experiments—WD, PD, and TK. Nanomaterials synthesis—WS, LN, JT, and KM. Nanomaterial characterization—A-LT and VB. Principal investigators—TK and JT.

## Conflict of Interest Statement

TK and JT are founders and shareholders in Acelerox, LLC. WD, LN, WS, PD, A-LT, JT, and TK are inventors on various approved and pending patents related to carbon nanomaterial synthesis and therapeutic use.
